# Is there a body of evidence for the treatment of patients with Adolescent Idiopathic Scoliosis (AIS)?

**DOI:** 10.1186/1748-7161-2-19

**Published:** 2007-12-31

**Authors:** Hans-Rudolf Weiss

**Affiliations:** 1 Asklepios Katharina Schroth, Spinal Deformities Rehabilitation Centre, Korczakstrasse 2, D-55566 Bad Sobernheim, Germany

## Abstract

Historically, the treatment options for AIS, the most common form of scoliosis are; exercises; in-patient rehabilitation; braces and surgery. While there is evidence in the form of prospective controlled studies that Scoliosis Intensive Rehabilitation (SIR) and braces can alter the natural history of the condition, there is no prospective controlled study comparing the natural history with surgical treatment.

One aim of the Scoliosis Society (SOSORT) should be; to help develop a body of research regarding the outcomes of conservative and operative treatment as well, and to highlight the problems of treatment indications in patients with AIS and other spinal deformities. Another aim is to help to improve the safety of patients who have surgery. By producing evidence-based information that can be used to develop guidelines that could aid both professionals and patients in making decisions about surgical and conservative options.

Although 'Scoliosis' is the official journal of the SOSORT and is the main forum for experts in the field of conservative management of patients with spinal deformities, there needs to be more wide spread attempt to develop a fuller body of evidence focussing on spine surgery as well.

## Editorial

Today evidence based medicine (EBM) and evidence based practice (EBP) are valuable instruments in the decision making process of professionals in the medical field. Restrictions upon resources of social health care systems have lead to calls for greater efficiency and cost effectiveness of treatment programmes. Therefore good quality evidence studies providing the highest of research are necessary to evaluate effectiveness of treatments.

The Centre for Evidence Based Medicine (EBM) [1] provides guidelines to spread the knowledge about EBM and its use. There is a special hierarchy of evidence based knowledge:

1. Smallest evidence is provided by "expert opinion"

2. Case reports/case series

3. Un-controlled studies

4. Controlled studies

5. Randomized controlled studies (RCT) and

6. Meta analyses from RCT

The quality and types of evidence help to segregate research into levels. They are graded (IV [lowest] – I [highest]) and from those levels recommendations for treatment are derived (Grade D [lowest] – Grade A [highest]).

Grade B recommendations for conservative treatment of scoliosis are justified. There are prospective controlled studies (level II) [2-4] and enough data from level III or IV, which are generally consistent [5] when taking into account studies from central Europe or Asia [6-9]. These levels of evidence seem not to have been reached in the United States [10,11].

Although randomised controlled trials (RCT's) provide the highest evidence the application of this study design is unrealistic for complex disorders like scoliosis. While pharmacological studies are the main field for RCT's until now no RCT on treatment outcomes for scoliosis is available.

In pharmacological studies one can easily standardise the treatments (drugs) to be investigated. Body weight of the patients and dosage of drugs can easily be measured [12].

**Figure 1:**
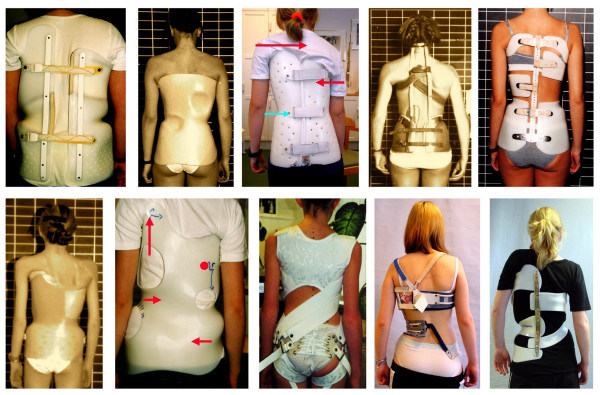
Very many braces are on the market today making it difficult to design studies on that topic because of the high variability of standards applied.

Scoliosis on the other hand is not a uniform condition. Even the subset of patients suffering from Adolescent Idiopathic Scoliosis (AIS) appears to include multiple variations in curve pattern, maturity, curve stiffness and sexual differences all influencing the outcome of treatment [13]. Recently claims have been made for an RCT on bracing [11,14,15], but the question remains to be answered; what brace, with what set amount of time, should be monitored and in which particular patient? It seems even difficult to define what exactly may be referred to as a "brace" as there is a wide variability of applications (Fig. 1). Treatment and subject treated are of such high variability that an RCT for bracing seems to be a very complex task.

Additionally to that, there is evidence on level II for the use of the Boston brace [3,4]. In the SRS multi-centre prospective controlled study [3] the survival analysis clearly has shown that the Boston brace was superior to observation only and to electrical stimulation. A long term controlled  prospective follow-up has also provided evidence that the Boston brace efficiently stops curvature progression [4]. A Meta-analysis [16] clearly unveils that brace wearing time is one determinant for a successful outcome. But if the brace could not influence the curvature, why would the amount of time spent wearing the brace be an important issue?

In the light of this evidence already available, an RCT is not only a complex task but an unethical one. To allow growing patients to continue without conservative treatment (a control group) until nothing except surgical intervention can help them, is completely unethical. Especially when one considers the problems with surgery, such as; primary risks; a re-surgery rate, which might be higher than 30% in the long-term [17-19] and future complications [17]. This type of approach cannot be regarded as patient-oriented. This is why the SOSORT offers clinicians and scientists to take part in prospective controlled studies on bracing. Within this society there is a unique opportunity to test different bracing approaches against each other in order to find the "Best Practice" of bracing. This will enable clincians in the near future the opportunity to give their conservatively treated patients the best possible advice and offer the best possible treatment in a more standardized way. Research on living patients should only be done in order to develop a useful treatment, this is why we need to be able to measure brace quality. We know that in-brace correction and compliance are the two main determinants of outcome [16,20,21]. Therefore in-brace correction might serve as a measure for brace quality and compliance as a measure for quality of management. Efforts have to be made to improve both of these.

**Figure 2:**
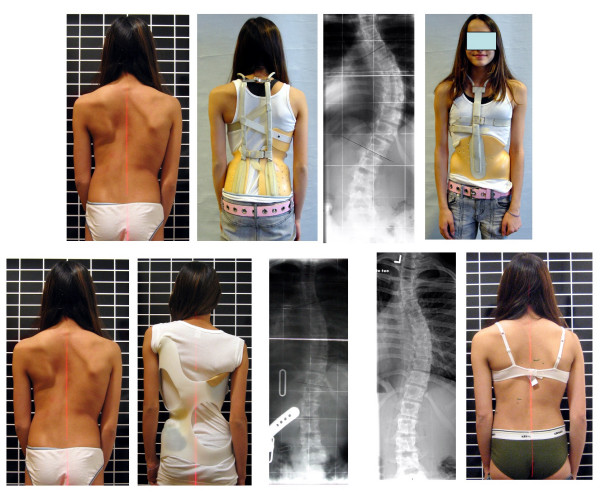
Patient with a thoracic curve of 56° corrected to 53° in her Milwaukee brace adjusted in North America (upper line). With this little correction effect one can predict no favourable outcome after this treatment. The question to be asked is: Is this patient a non-responder? In a brace of the Chêneau type she gained an in-brace correction down to 27° and after 15 months of treatment she clinically improved and her curve (without the brace) was 36° (lower line).

Unfortunately many studies on bracing, mainly coming from the US, do not attempt to find ways to improve this measurement [10,11,14,22]. Whether a brace works or not seems to depend upon the fate of the individual patient and not on brace quality. Some SRS Surgeons introduced the term "brace responder" or "non-responder" [23] as if it was the patients fault when there is no successful outcome. No one attempts to explain why some patients are "non-responders" and with another brace the same patients are "responders" [24] (Fig. 2).

Examining the evidence at a deeper level in recent publications that claim RCT's on bracing [11,14,15]:

Dolan et al. [11] have found a wide variation of surgical rates for a number of brace types worn 8 to 20 hours per day. The variation in the pool of selected data ranged from a surgical rate of 1% to 43%. After calculating this data the authors concluded: *"Based on the evidence presented here, one cannot recommend one approach [bracing] over the other [observation] to prevent the need for surgery in AIS" *[11]

But there are four main weaknesses within this study; the subject (a brace) and the outcome parameter (rate of surgery), are components which are known to contain great ranges of variability and therefore, the only justified conclusion to be drawn from this study would be that 'rate of surgery' cannot be used to generate valid evidence.

Secondly, the authors set the inclusion and exclusion criteria in a way to enable the exclusion of studies that have presented a smaller rate of surgery and also those with the additional use of physiotherapy. Only one [6] of the four studies from outside the US [6-9] covering this topic is cited (but not included) in their paper, due to their specific choice of inclusion and exclusion criteria.

As Hawes clearly highlighted [25], a large number of papers from the US containing an undefined number of patients treated by physiotherapy do exist, even in natural history studies. But as physiotherapy historically has been recognized as 'no treatment' [25] they tend not to be considered within research reviews.

The third main problematic area within this study is that there is no mention of the correction effect of the braces used in this study. When brace quality is not assessed and in-brace correction is the only quality parameter applied, no conclusions about the use of bracing can be made. Braces from Europe have not been included in this paper, although they are of higher standards in terms of in-brace correction [13,20,21,24,26].

The final and most significant weakness of this study is in relation to 'a need for surgery' for patients with AIS [11]. As signs and symptoms of scoliosis in AIS patients cannot be cured by surgery [27], there is no indication for surgery other than the cosmetic indication. As Goldberg pointed out in her paper [10], which can be viewed as the precursor of the paper by Dolan and Weinstein [11], surgery replaces one pathology (a curved spine) with another (a stiff spine). Therefore, there are no clinical indications for surgery in patients with AIS, when we are dealing with a relatively benign condition [28,29] and when there is no proof that surgery changes signs and symptoms of scoliosis [27].

In another paper by Dolan et al [14], the subject of the 'professional' opinion concerning the effectiveness of bracing was investigated. As could have been expected, a high variability in opinions has been found in the group of surgeons and a small number of other professions. In this study a RCT on braces has also been postulated. One question remains to be answered: Is a surgeon automatically a 'professional' when bracing is the specialised subject?

**Figure 3:**
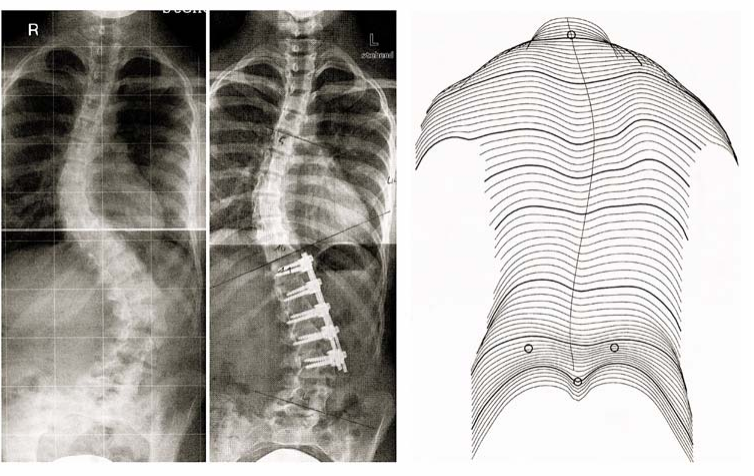
On the left X-rays before and after surgery and on the right clinical appearence after surgery. The patient is unbalanced. Due to the short instrumentation area there is a sharp angle at the junctional lumbo-pelvic area making it reasonable to assume that a reoperation will be necessary some day.

**Figure 4:**
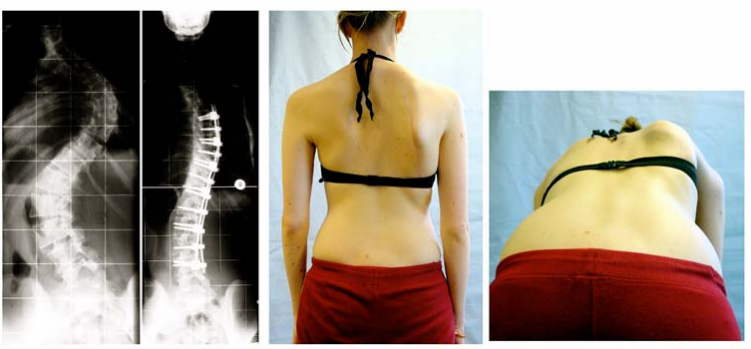
After surgery there is still a significant rib-hump visible.

One might assume that the main benefit of surgery is correction. But when one realises that primary correction effects are not necessarily stable after surgery [30-40], not even in the first year and that neither back shape nor self esteem have been corrected to a satisfactory level by the surgical intervention [41] and that a balanced appearance of a patient is not necessarily the outcome of surgical intervention (Fig. 3 and 4), a more scientific basis remains to be desired on the true outcome after surgery.

With respect to surgery, there are no level II or level I studies to support the use of surgery in the treatment of AIS and as previously stated by Hawes [27], signs and symptoms of AIS cannot be changed by surgery.

But respect should remain for the psychological indication for surgery when a patient with AIS is unable to cope with the deformity.

The assumption that there is an indication for surgery', in spite of the known long-term risks [17-19,27], however should be made cautiously by those with an ethical responsibility to the patient as it seems that this lacks a scientific evidence base.

**Figure 5:**
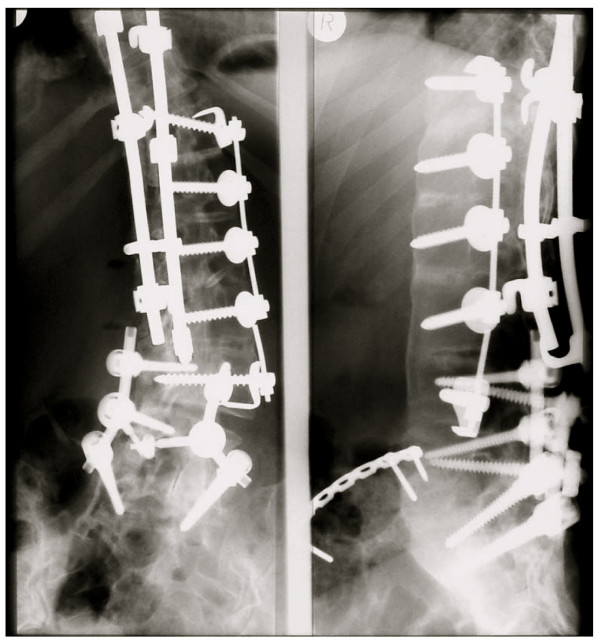
Condition after ventrodorsal reoperation. Stabilisation seemed necessary down to the sacrum. As there are no free segments below the fusion area problems may arise in the future.

The long-term risks of surgical scoliosis treatment are not conclusively agreed upon. No studies are available to describe the aging operated patient with AIS, but when one considers that the 10 to 20 year risk for a re-operation (Fig. 5) may already be as high as 29% [19], the real long-term risks have to be estimated at > 30% [27], it is this fact the patient should be informed of before consenting to undergo this procedure, as consent should always be an informed decision [18].

To help to develop the body of research regarding the outcome of surgery and to highlight the problems of treatment indications in patients with AIS and other spinal deformities we would like to open the *Scoliosis *Journal to papers that discuss surgical procedures.

One of our aims is to improve patients' safety in surgery by producing evidence-based information that can be used to develop guidelines that could aid both professionals and patients in making decisions about surgical and conservative options.

Within this society we have well known spinal surgeons who are specialists in conservative management of scoliosis as well. This is why I am confident that to include papers with surgical content, is a step towards an equilibrated and balanced view on scoliosis management.

Tomasz Kotwicki, deputy editor in charge will oversee papers with surgical content from now on.

My very best wishes to my friend and colleague Dr. Tomasz Kotwicki, who surely is both: a dedicated physician and a remarkable pediatric spine surgeon.
